# Cell segmentation methods for label-free contrast microscopy: review and comprehensive comparison

**DOI:** 10.1186/s12859-019-2880-8

**Published:** 2019-06-28

**Authors:** Tomas Vicar, Jan Balvan, Josef Jaros, Florian Jug, Radim Kolar, Michal Masarik, Jaromir Gumulec

**Affiliations:** 1 Department of Biomedical Engineering, Faculty of Electrical Engineering and Communication, Brno University of Technology, Technicka 3058/10, Brno, CZ-61600 Czech Republic; 2 Department of Physiology, Faculty of Medicine, Masaryk University, Kamenice 5, Brno, CZ-62500 Czech Republic; 3 Department of Pathological Physiology, Faculty of Medicine, Masaryk University, Kamenice 5, Brno, CZ-62500 Czech Republic; 4 Central European Institute of Technology, Brno University of Technology, Purkynova 656/123, Brno, CZ-612 00 Czech Republic; 5 Max Planck Institute of Molecular Cell Biology and Genetics, Pfotenhauerstr. 108, Dresden, DE-01307 Germany; 6 Department of Histology and Embryology, Faculty of Medicine, Masaryk University, Kamenice 5, Brno, CZ-62500 Czech Republic; 7 International Clinical Research Center, St. Anne’s University Hospital, Pekarska 664/53, Brno, CZ-65691 Czech Republic

**Keywords:** Microscopy, Cell segmentation, Image reconstruction, Methods comparison, Differential contrast image, Quantitative phase imaging, Laplacian of Gaussians

## Abstract

**Background:**

Because of its non-destructive nature, label-free imaging is an important strategy for studying biological processes. However, routine microscopic techniques like phase contrast or DIC suffer from shadow-cast artifacts making automatic segmentation challenging. The aim of this study was to compare the segmentation efficacy of published steps of segmentation work-flow (image reconstruction, foreground segmentation, cell detection (seed-point extraction) and cell (instance) segmentation) on a dataset of the same cells from multiple contrast microscopic modalities.

**Results:**

We built a collection of routines aimed at image segmentation of viable adherent cells grown on the culture dish acquired by phase contrast, differential interference contrast, Hoffman modulation contrast and quantitative phase imaging, and we performed a comprehensive comparison of available segmentation methods applicable for label-free data. We demonstrated that it is crucial to perform the image reconstruction step, enabling the use of segmentation methods originally not applicable on label-free images. Further we compared foreground segmentation methods (thresholding, feature-extraction, level-set, graph-cut, learning-based), seed-point extraction methods (Laplacian of Gaussians, radial symmetry and distance transform, iterative radial voting, maximally stable extremal region and learning-based) and single cell segmentation methods. We validated suitable set of methods for each microscopy modality and published them online.

**Conclusions:**

We demonstrate that image reconstruction step allows the use of segmentation methods not originally intended for label-free imaging. In addition to the comprehensive comparison of methods, raw and reconstructed annotated data and Matlab codes are provided.

**Electronic supplementary material:**

The online version of this article (10.1186/s12859-019-2880-8) contains supplementary material, which is available to authorized users.

## Background

Microscopy has been an important technique for studying biology for decades. Accordingly, fluorescence microscopy has an irreplaceable role in analyzing cellular processes because of the possibility to study the functional processes and morphological aspects of living cells. However, fluorescence labeling also brings a number of disadvantages. These include photo-bleaching, difficult signal reproducibility, and inevitable photo-toxicity (which results not only from staining techniques but also from transfection) [1]. Label-free microscopy techniques are the most common techniques for live cell imaging thanks to its non-destructive nature, however, due to the transparent nature of cells, methods of contrast enhancement based on phase information are required.

The downside of contrast enhancement is an introduction of artifacts; Phase contrast (PC) images contain halo and shade-off, differential image contrast (DIC) and Hoffman Modulation Contrast (HMC) introduce non-uniform shadow-cast artifacts (3D-like topographical appearance). Although various segmentation procedures have been developed to suppress these artifacts, a segmentation is still challenging.

On the other hand, quantitative phase imaging (QPI), provides artifact-free images of sufficient contrast. Although there are no standardized methods for the segmentation of QPI-based images, fundamental methods for segmentation of artifact-free images (e.g. from fluorescence microscopy) will be utilized.

In this review, we describe and compare relevant methods of the image processing pipeline in order to find the most appropriate combination of particular methods for most common label-free microscopic techniques (PC, DIC, HMC and QPI). Our aim is to evaluate and discuss the influence of the commonly used methods for microscopic image reconstruction, foreground-background segmentation, seed-point extraction and cell segmentation. We used real samples - viable, non-stained adherent prostatic cell lines and captured identical fields of view and cells manually segmented by a biologist. Compared to microscopic organisms like yeast or bacteria, adherent cells are morphologically distinctly heterogeneous and in label-free microscopy, the segmentation is therefore still a challenge. We will use the most common imaging modalities used by biologist and we will provide a recommendation of image processing pipeline steps for particular microscopic technique.

The segmentation strategies tested herein are selected to provide the most heterogeneous overview of recent state of the art excluding the simplest and outdated methods (e.g. simple connected component detection, ultimate erosion, distance transform without h-maxima etc.). Deep-learning strategies are intentionally not included due to their distinct differences, high demands on training data and the range of possible settings (training hyperparameters, network architecture, etc.).

## Results

**Fig. 1:**
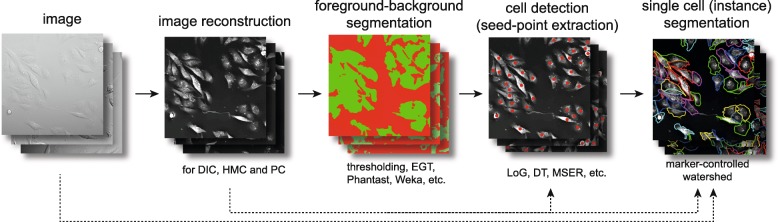
Block diagram showing segmentation approach. For details of individual steps, see Results and Materials and Methods.EGT, empirical gradient treshold; LoG, Laplacian of Gaussians, DT, distance transform, MSER maximally stable extremal region

In the paragraphs below we provide a detailed summary of each image processing step from the pipeline (see Fig. 1), followed by short description of achieved results. We start with description of “all-in-one” tools and continue with image reconstruction, foreground-background segmentation, cell detection and final single cell segmentation (i.e. instance segmentation).

Due to the large number of tested methods and approaches, we have decided to introduce a specific designation of the methods. We used prefix in order to refer to image reconstruction (‘r’), foreground-background segmentation (‘s’) and cell detection (‘d’) and finally to all-in-one tools (‘aio’). The list of these designations, number of parameters to be adjusted in these methods and computational demands are provided in Table 1.

**Table 1:** List of tested segmentation methods and all-in-one segmentation tools and definition of abbreviations

Segmentation step	Abbreviation	Description	Setable parameters	Computational time	Ref.
All in one tools					
	aioFasright	Nucleus editor of Farsight toolkit	N/A	4.96 s	[2]
	aioCellX	segmentation, fluorescence quantification, and tracking tool CellX	N/A	10.30 s	[3]
	aioFogbank	single cell segmentation tool FogBank according Chalfoun	N/A	12.00 s	[4]
	aioFastER	fastER - user-friendly tool for ultrafast and robust cell segmentation	N/A	0.42 s	[5]
	aioCellProfiler	tool for cell analysis pipelines including single cell segmentation	N/A	11.8 s	[10]
	aioDMGW	Dry mass-guided watershed method, (Q-PHASE, Tescan)		1.00 s	
Reconstruction					
	rDIC-Koos	DIC/HMC image reconstruction according Koos	2	36.60 min	[12]
	rDIC-Yin	DIC/HMC image reconstruction according Yin	2	2.10 s	[13]
	rPC-Yin	PC image reconstruction according Yin	4	13.10 min	[14]
	rPC-Tophat	PC image reconstruction according Thirusittampalam and Dewan	1	0.17 s	[15, 16]
Foreground-background segmentation					
	sST	simple thresholding	1	<0.01 s	
	sOtsu	thresholding using Gaussian distribution	0	<0.01 s	[17]
	sPT	thresholding using Poisson distribution	0	<0.01 s	[2]
	sEGT	empirical gradient threshold	3	0.24 s	[18]
	sPC-Juneau	Feature extraction approach according Juneau	3	0.26 s	[19]
	sPC-Topman	Feature extraction approach according Topman	4	0.35 s	[20]
	sPC-Phantast	Phantast toolbox developed by Jaccard	5	0.35 s	[21]
	sLS-Caselles	Level-set with edge-based method	2	31.40 s	[22]
	sLS-ChanVese	Level-set with region-based method	2	11.10 s	[23]
	sGraphCut	Graph-Cut applied on recosntructed and raw data	2	15.80 s	[24]
	sWekaGraphCut	Graph-Cut applied on probability maps generated by Weka	2	31.80 min**	[24]
	sIlastikGraphCut	Graph-Cut applied on probability maps generated by Ilastik	2	31.52 min**	[24]
	sIlastik	machine learning tool by Sommer	N/A	31.20 min+21 s*	[25].
	sWeka	machine learning tool by Arganda-Carreras	N/A	27.60 min+2.20 min*	[26]
Cell detection (seed-point extraction)					
	dLoGm-Peng	multiscale LoG by Peng	4	3.60 s	[27]
	dLoGm-Kong	multiscale LoG by Kong	5	4.20 s	[28]
	dLoGg-Kong	generalized LoG filter by Kong	2	46.40 s	[28]
	dLoGg-Xu	generalized LoG filter by Xu	3	5.10 s	[29]
	dLoGh-Zhang	Hessian analysis of LoG images by Zhang	1	8.90 s	[30]
	dFRST	fast radial-symmetry transform	5	153.10 s	[31]
	dGRST	generalized radial-symmetry transform	5	572.30 s	[32]
	dRV-Qi	radial voting methods by Qi et al.	5	95.00 s	[33]
	dDT-Threshold	distance transform by Thirusittampalam, threshold-generated foreground	4	0.11 s	[15]
	dDT-Weka	distance transform by Thirusittampalam, sWeka-generated foreground	3	0.11 s *‡*	[15]
	dMSER	maximally stable extremal region method (MSER)	3	2.10 s	[34]
	dCellDetect	machine learning method based on MSER	1	141.70 s/60.20 s*	[35]
Single cell (instance) segmentation					
	MCWS *†*	Marker-conttrolled watershed	0	1.40 s	
	MCWS-dDT *†*	Marker-conttrolled watershed on DT image	0	1.41 s	

For detailed list of optimized parameters see Additional file 1. * computational time for learning based approaches indicated as two values for learning and classification. ** computational time for Weka+Graph cut combination shown as sum time of these methods. *‡* not includes time for Weka probability map creation, *†* indicate final segmentation step following foreground-background segmentation and seed-point extraction. Number of parameters in “all-in-one” approaches not shown because of the GUI-based nature, similarly, not shown for learning-based approaches, see Methods section for details. Computational time shown for one 1360 ×1024 DIC field of view

### “All-in-one” tools

First, we performed an analysis with the available commercial and freeware “all-in-one” tools including FARSIGHT [2], CellX [3], Fogbank [4], FastER [5], CellTracer [6], SuperSegger [7], CellSerpent [8], CellStar [9], CellProfiler [10] and Q-PHASE’ Dry mass guided watershed (DMGW) [11]. As shown in Table 2 the only algorithm providing usable segmentation results for raw images is Fogbank, which is designed to be an universal and easy to set segmentation tool. Very similar results were provided by CellProfiler, which is easy to use tool allowing to crate complete cell analysis pipelines, however, it works sufficiently only for reconstructed images. The QPI’ dedicated DMGW provided exceptional results, but for this microscopic technique only. The remaining methods did not provide satisfactory results on label free data; FastER, although user-friendly, failed because of the nature of its maximally stable extremal region (MSER) detector. FARSIGHT failed with the automatic threshold during foreground segmentation. CellX failed in both the cell detection with gradient-based Hough transform and in the membrane pattern detection because of indistinct cell borders. The remaining segmentation algorithms - CellStar, SuperSegger, CellSerpent - were completely unsuitable for label-free non-round adherent cells with Dice coefficient <0.1 and thus are not listed in Table 2 and Fig. 4.

**Table 2:** The segmentation efficacy (shown as Dice coefficient) of individual segmentation steps on raw and reconstructed image data

Method	Segmentation efficacy (Dice coefficient)									
	QPI	DIC			HMC			PC		
	raw	raw	rDIC Koos [12]	rDIC Yin [13]	raw	rDIC Koos [12]	rDIC Yin [13]	raw	rPC Yin [14]	rPC TopHat [15]
Foreground-background segmentation										
sWekaGraphCut	0.96	0.86	0.89	0.84	0.86	0.84	0.84	0.86	0.80	0.84
sIllastikGraphCut	0.94	0.87	0.89	0.84	0.87	0.84	0.84	0.80	0.80	0.84
sWeka	0.94	0.85	0.87	0.80	0.85	0.82	0.79	0.81	0.72	0.81
sIlastik	0.94	0.85	0.86	0.80	0.82	0.82	0.79	0.84	0.72	0.82
sLS-Caselles	0.88	0.83	0.82	0.79	0.84	0.79	0.79	0.77	0.75	0.79
sEGT	0.89	0.88	0.85	0.64	0.86	0.79	0.70	0.74	0.68	0.79
sPC-Phantast	N/A	N/A	N/A	N/A	N/A	N/A	N/A	0.77	N/A	N/A
sPC-Juneau	0.85	0.85	0.84	0.59	0.82	0.77	0.69	0.73	0.72	0.76
sPC-Topman	N/A	N/A	N/A	N/A	N/A	N/A	N/A	0.72	N/A	N/A
sLS-ChanVese	0.61	0.48	0.74	0.55	0.68	0.67	0.36	0.64	0.65	0.76
sGraphCut	0.92	0.38	0.78	0.64	0.32	0.59	0.58	0.40	0.70	0.74
sST	0.92	0.339	0.76	0.61	0.31	0.72	0.53	0.40	0.69	0.73
sPT	0.83	0.34	0.60	0.34	0.30	0.46	0.08	0.29	0.67	0.73
sOtsu	0.62	0.34	0.36	0.31	0.28	0.16	0.18	0.24	0.51	0.66
Cell detection (seed point extraction)										
dGRST	0.94	0.65	0.79	0.85	0.75	0.81	0.85	0.81	0.77	0.88
dLoGm-Kong	0.90	0.83	0.90	0.86	0.72	0.84	0.85	0.52	0.69	0.78
dFRST	0.94	0.58	0.78	0.82	0.70	0.78	0.82	0.82	0.74	0.88
dLoGm-Peng	0.89	0.71	0.86	0.78	0.69	0.83	0.86	0.51	0.73	0.84
dLoGg-Kong	0.85	0.83	0.80	0.84	0.74	0.82	0.83	0.43	0.72	0.79
dDT-Weka	0.81	0.68	0.81	0.74	0.73	0.72	0.75	0.80	0.76	0.78
dLoGg-Xu	0.84	0.77	0.80	0.80	0.65	0.81	0.78	0.52	0.71	0.78
dDT-Threshold	0.94	0.26	0.91	0.86	0.54	0.86	0.84	0.49	0.76	0.81
dRV-Qi	0.77	0.61	0.57	0.58	0.70	0.48	0.48	0.82	0.59	0.65
dMSER	0.93	0.06	0.55	0.58	0.29	0.82	0.69	0.65	0.79	0.68
dCellDetect	0.92	0.00	0.88	0.89	0.00	0.83	0.84	0.00	0.71	0.81
dLoGh-Zhang	0.82	0.13	0.52	0.64	0.25	0.63	0.65	0.49	0.70	0.61
Single cell (instance) segmentation										
MCWS-dDT *†*	0.77	0.58	0.66	0.61	0.47	0.54	0.55	0.52	0.37	0.62
MCWS *†*	0.82	0.55	0.69	0.63	0.26	0.54	0.53	0.41	0.39	0.60
aioFogbank	0.71	0.54	0.55	0.42	0.44	0.38	0.39	0.46	0.34	0.19
aioCellProfiler	0.69	0.37	0.55	0.38	0.19	0.45	0.27	0.09	0.41	0.54
aioDMGW	0.82	0.08	0.62	0.38	0.00	0.48	0.29	0.10	0.39	0.65
aioFasright	0.21	0.15	0.43	0.00	0.00	0.26	0.14	0.03	0.37	0.57
aioCellX	0.34	0.03	0.08	0.21	0.02	0.18	0.05	0.07	0.03	0.16
aioFastER	0.09	0.03	0.07	0.00	0.02	0.17	0.01	0.25	0.08	0.06

Sorted by Dice coefficient (high to low). N/A, not applicable, for foreground background segmentation, methods designated for PC image were not deployed on other microscopic modalities

Because of the low segmentation performance of the examined “all-in-one” methods, we decided to divide the segmentation procedure into four steps - (1) image reconstruction (2) background segmentation, (3) cell detection (seed expansion) and (4) segmentation tailored to the specific properties of individual microscopic techniques (see Fig. 1).

### Image reconstruction

As shown, the performance of most “all-in-one” methods is limited for label-free data, in particular due to the presence of contrast-enhancing artifacts in microscopic images. Image reconstruction was therefore employed to reduce such artifacts. Methods by Koos [12] and Yin [13] (further abbreviated rDIC-Koos and rDIC-Yin, respectively) were used for DIC and HMC images. Images of PC microscopy were reconstructed by Top-Hat filter involving algorithm by the Dewan [16] (rPC-TopHat), or Yin method (rPC-Yin) [14].

**Fig. 2:**
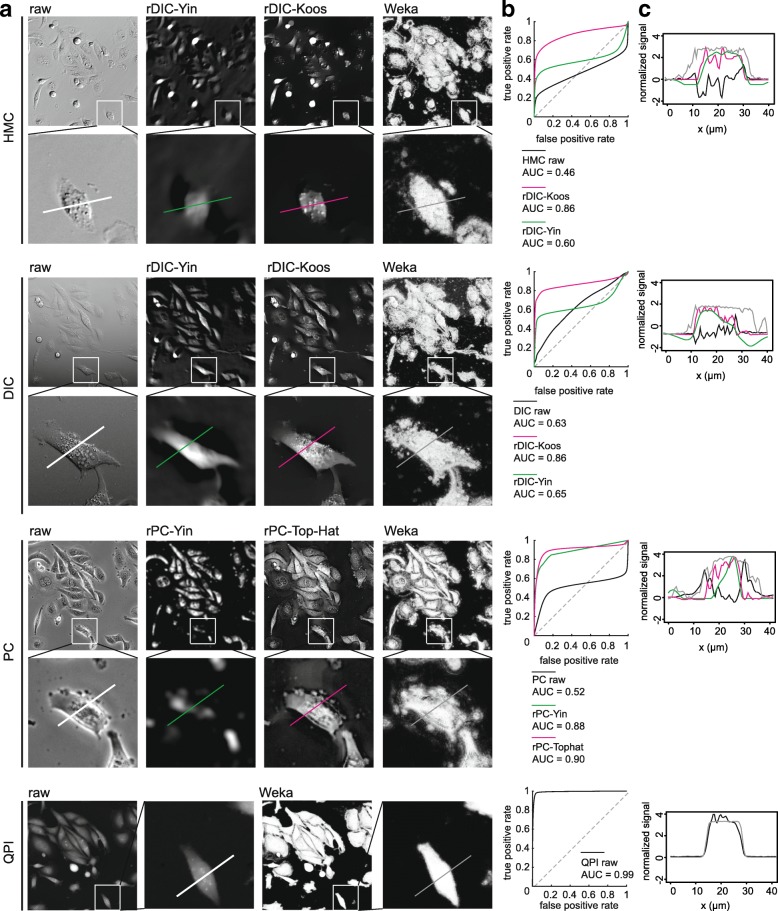
Quality of reconstructions a. field of view for raw and reconstructed HMC, DIC, PC and QPI images. Image width is 375 μm and 85 μm for field of view and detail below (**b**). receiver operator curve for particular image reconstruction (**c**). profile of reconstructed image corresponding to section in detail in (**a**). AUC, area under curve, ROC, receiver-operator curve
- The AUC of QPI was distinctly higher with values near 0.99
- Computationally more-demanding methods (rDIC-Koos and rPC-Yin) perform better except for relatively simple rPC-Top-Hat, which provides similar results
- Probability maps generated by sWeka or sIllastik can be used like reconstructions in later segmentation steps. The advantage of this approach is the absence of the need to optimize parameters.

Generally, following conclusions apply for image reconstructions:

- No distinctive differences in image reconstruction efficacy were observed between the microscopic methods apart from QPI, as shown in Fig. 2 (described by area under curve, AUC, see Methods for details).

#### DIC and HMC reconstructions

With regard to the morphology of reconstructed images, rDIC-Koos provides a detailed structure of the cells with distinctive borders from the background. For rDIC-Yin [13], details of the reconstructed cells are more blurred and uneven background with the dark halos around the cells (see Fig. 2) complicating the following segmentation. As a result, AUC of rDIC-Yin was distinctly lower as compared with the others.

Both rDIC-Koos [12] and rDIC-Yin [13] methods work on the principle of minimizing their defined energy function. The main difference is that better-performing Koos [12] uses l1-norm (instead of l2) for sparse regularization term. Yin’s l2-norm, on the other hand, enables derivation of closed form solution, which is much simpler and thus faster to compute. Time needed for the reconstruction is dramatically different - 2.1 s, 36.6 min, 13.1 min and 0.17 s for rDIC-Koos, rDIC-Yin, rPC-Koos and rPC-TopHat, respectively. rDIC-Koos also introduces a parameter for the number of iterations, which is however insensitive within the tested range.

Although these methods were not designed for use on HMC images, the same conclusions also apply for the reconstruction of those images, which showed only slightly worse results. The results of reconstruction accuracy can be seen in Fig. 2. Combinations of the best-performing parameters are listed in the Additional file 1.

#### Phase contrast reconstruction

From the perspective of cellular morphology of reconstructed images, rPC-TopHat creates artifacts between closely located cells with the borders precisely distinguishable. Reconstruction based on rPC-Yin [14] causes an even background without observable artifacts around the cells, however cell borders are missing and mitotic cells are not properly reconstructed (see Fig. 2).

The optimization of the PSF parameters of rPC-Yin reconstruction is problematic. The PSF parameters of a particular microscope are not always listed or known. Searching for these parameters with optimization proved to be complicated. Because the optimizing function is not smooth and contains many local local extrema, the result changes significantly and chaotically even with a small change of parameters or, at the same time, combinations of parameter settings give very similar (near optimal) results.

Regarding the computational times, the rPC-Yin reconstruction works very similarly as the rDIC-Koos approach for DIC, with similar computational difficulties. The result of a simple top-hat filter unexpectedly turned out to be comparable to the complex and computationally difficult rPC-Yin method. For the reconstruction performance see Fig. 2, for optimal parameter setting see the Additional file 1.

### Foreground-background segmentation

In the next step of the workflow, the image foreground (cells) was segmented from the image background. Both unprocessed and reconstructed images were used. Following strategies were used for the foreground-background segmentation: (a) Thresholding-based methods: simple threshold (sST), automatic threshold based on Otsu et al. [17] (sOtsu), and Poisson distribution-based treshhold (sPT) [2], (b) feature-extracting strategies: empirical gradient threshold (sEGT) [18] and approaches specific for PC microscopy by Juneau et al. (sPC-Juneau) [19], Jaccard et al. (sPC-Phantast) [21], and Topman (sPC-Topman) [20]), (c) Level-Set-based methods: Castelles et al. [22] (sLSCaselles), and Chan-Vese et al. [23] (sLS-ChanVese), (d) Graph-cut [24], and (e) Learning-based Ilastik [25], and Trainable Weka Segmentation [26].

**Fig. 3:**
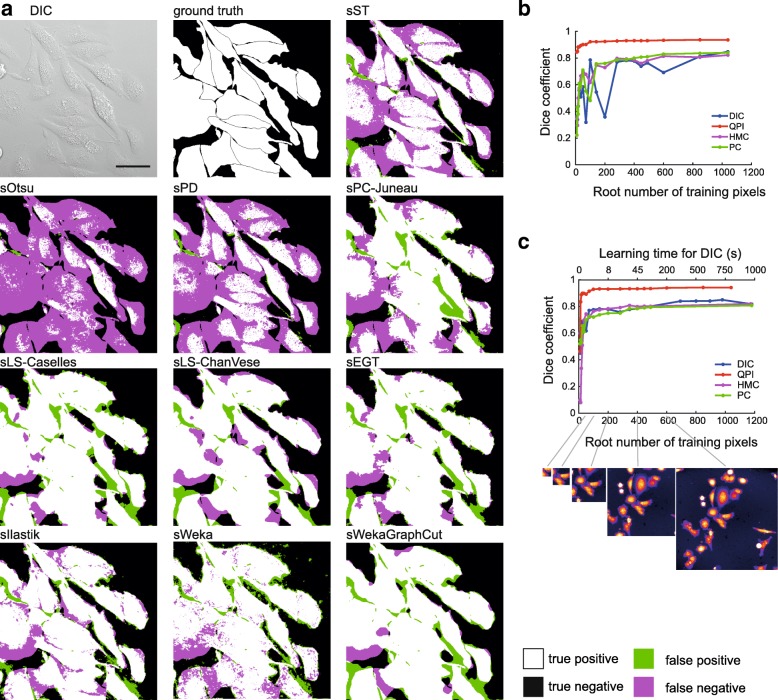
Foreground-background segmentation step. **a** representative images showing tested foreground-background segmentation methods of rDIC-Koos-reconstructed DIC image. Dependency between area used for training and Dice coefficient for learning-based approach Ilastik (**b**) and Weka (**c**). scalebar indicates 50 μm

Based on the obtained results, this step can be considered the least problematic in segmentation, with the following general findings:

- Well-performing methods (e.g. sWeka, sIllastik, sLS-Caselles,sEGT, sPC-Juneau) are robust enough to work even on unreconstructed data.
- Image reconstruction improves foreground-background segmentation efficacy and once reconstructed, there are no distinct differences in segmentation efficacy between microscopic techniques
- QPI performs dramatically better even unreconstructed
- Learning-based methods (sWeka and sIlastik) perform better by a few units of percents. Its performance can further be improved with GraphCut.
- More time-consuming methods (sLS-Caselles, sLS-ChanVese, sGraphCut, sWeka, sIlastik) does not necesarily provide better results. For detailed results, see chapters below and Fig. 3.

#### Threshold-based approaches

The Simple threshold (sST) provides better results than automatic thresholding techniques assuming Poisson distribution (sPT) or Otsu method (sOtsu). The potential of these automatic techniques lies in the segmentation of images, where optimal threshold value varies between the images. However, this is not necessary for QPI images (constant background value increases success of sST) and for reconstructed images with background removal (background values are close to zero, so the histogram cannot be properly fitted with Gaussian or Poison distribution, see Table 2). There are not any parameters to optimize for sOtsu and sPT methods, which is the main advantage. The results of thresholding could be potentially improved by morphological adjustments. Regarding the computational times, these are the simplest and thus the fastest possible methods, which are listed mainly to provide basic idea about the segmentability of our data.

#### Feature-extraction-based approaches

The feature-based approaches - sEGT, sPC-Topman, sPC-Phantast and sPC-Juneau are all mainly based on the extraction of some feature image, which is then thresholded and morphologically modified. Because of feature thresholding strategies, the segmentation is possible without the image reconstruction. Thus these methods are among the most straightforward approaches to extract and threshold some local features (e.g. absolute value of gradient or local standard deviation).

All these methods can be easily adjusted, have the same number of parameters and the segmentation performance is very similar (see Table 1) with slightly better-performing sEGT. Compared to the other feature-extraction-based methods, sEGT includes elimination of small holes.

The performance of feature-extraction methods is technique-dependent with the highest scores for DIC and QPI and the lowest (but still high) for PC. This is mostly due to halos in PC; although sPC-Topman and sPC-Phantast are extended by the elimination of PC artifact regions, sPC-Topman have even worse results than sEGT or sPC-Juneau and sPC-Phantast leads to a slight improvement only for a cost of more parameters to be set.

From feature thresholding methods, sEGT was shown to be the best with only a small number of parameters and great versatility. Because of its percentile based threshold, it can be used even with a default setting, which achieves e.g. 0.84 Dice coefficient value for QPI. Compared to threshold-based methods, feature-extraction strategies perform approximately 10% better. Considering the computational demands, these methods are very simple and fast - comparable to simple thresholding.

#### Level-set-based approaches

Both sLS-Caselles [22] and sLS-ChanVese [23] active contours tended to shrink too much, which was compensated by setting additional force to negative sign, which leads to a tendency of the contour to grow. The increase of the additional force leads to a better Dice coefficient value until a breaking point, after which it leads to the total divergence of the contour. Still, the value of additional force had a much greater influence than the smoothness parameter.

Compared to the above-mentioned foreground-background segmentation strategies, the level-set based methods are relatively complicated and computationally difficult (tens of seconds vs. less than 1 s per FOV, Table 1). In their basic forms, two parameters are needed to be set. Another great disadvantage is that proper initialization is required, mainly the sLS-Caselles method is very sensitive to initialization. Based on segmentation results, sLS-ChanVese is applicable on reconstructed images only, and does not even reach the segmentation efficacy of simple threshold results. On the other hand, sLS-Caselles is applicable on raw images, but only for PC images it surpasses the otherwise much faster sEGT.

#### Graph-cut

There is a large number of methods and modifications based on Graph-Cut. Herein, we tested the basic model only. When Graph-cut was employed on the reconstructed images (sGraphCut), the highest Dice coefficient was obtained among non-trainable approaches except for rPC-Tophat, being surpassed by sLS-ChanVese. Nevertheless, Graph-Cut does not outperform simple threshold dramatically, providing roughly 2% increase in Dice coefficient and is only suitable for reconstructed data.

Regarding differences between microscopic methods, the Graph-cut approach was most suitable for reconstructed DIC images, followed by PC and HMC. Regarding the computational times, this method performs similarly as the level-set-based strategies (tens of seconds per FOV - Tables 1 and 2). Optimized values are shown in Additional file 1.

#### Trainable approaches

Trainable Weka segmentation (sWeka) and Ilastik (sIlastik) were employed in this step. Similarly to the feature-extracting approaches, these are applicable on raw, unreconstructed data. Both sIlastik and sWeka outperformed all tested foreground-background segmentation methods with Dice coefficient up to 0.94 for QPI and up to 0.85 for DIC, HMC and PC.

Regardless of the imaging modality used, there was an identifiable “breakpoint” in the dependency between the area size used for learning and the segmentation efficacy after which no dramatic increase in Dice coefficient was observed, see Fig. 3. For DIC, PC, and HMC it was approx. at the size 70×70 px., for QPI, distinctly smaller area was necessary, approx. 25×25 px. These areas roughly correspond to the cell size. However, to demonstrate the theoretical maximum of this method and to compare it with Ilastik, learning from one whole FOV for DIC, HMC, and PC and from 3 FOVs for QPI was deployed (see Table 2.

Next, an effect of learning from one continuous area in one FOV, or smaller patches of same sizes from multiple FOVs was tested. On DIC data it was demonstrated that learning from multiple areas causes significant, but slight 2% increase increase in Dice coefficients.

No increase of Dice coefficient was observed when different filters were enabled apart from the set of default ones (“default” vs “all”) as well as changing of minimum/maximum sigma. This was tested with a random search approach and with the Dice coefficient varying ±0.01. Both Weka and Ilastik provide almost the same segmentation results and are identically time-demanding.

There are two parameters to be optimized: terminal weights and edge weight. Edge weight (designated as “smoothness” in the GUI, range 0-10) reflects a penalty for label changes in the segmentation (higher values cause smoother result).

Furthermore, probability maps generated by sWeka and Ilastik under optimal settings were exported and these maps were further segmented by Graph-Cut (sWekaGraphCut/sIlastikGraphCut) and optimized in a same manner as sGraphCut on reconstructed data. A slight increase of the segmentation efficacy caused the sWekaCraphCut/sIlastikCraphCut combination to be the most efficient foreground-background segmentation method for QPI, HMC, and PC, only being surpassed by EGT on raw DIC image data. More importantly, this was achieved without the need of the image reconstruction.

### Cell detection (seed-point extraction)

Once the foreground (cells) is separated from the background, the next step is to identify individual cells (seed points). The following strategies were used: (a) Cell shape-based, Laplacian of Gaussian (LoG) variants Peng et al. [27] (dLoGm-Peng), Kong et al.[28] (dLoGm-Kong), Hessian Zhang et al.[30] (dLoGh-Zhang), generalized Kong et al. [28] (dLoGg-Kong), generalized Xu et al. [29] (dLoGg-Xu), (b) Cell shape-based, generalized radial symmetry transform [32] (dGRST), fast radial symmetry transform [31] (dFRST), (c) Qi et al.[33] radial voting (dRV-Qi), (d) distance transform [15] (dDT-Threshold, dDT-Weka), (e) Maximally Stable Extremal Region [34] (dMSER), and (f) dCellDetect [35]. Following general conclusions are applicable for this segmentation step:

- Seed-point extraction is crucial step of cell segmentation
- The requirement of reconstructed images is a significant bottleneck of the seed-point extraction
- multiscale and generalized LoG are among the most robust and to some extent work also on unreconstructed data
- Radial symmetry transform-based strategies perform well
- Seed-point extraction is exceptional on QPI data
- Learning-based approach dCellDetect provide exceptional results on reconstructed data.

#### Laplacian of Gaussian-based strategies

**Fig. 4:**
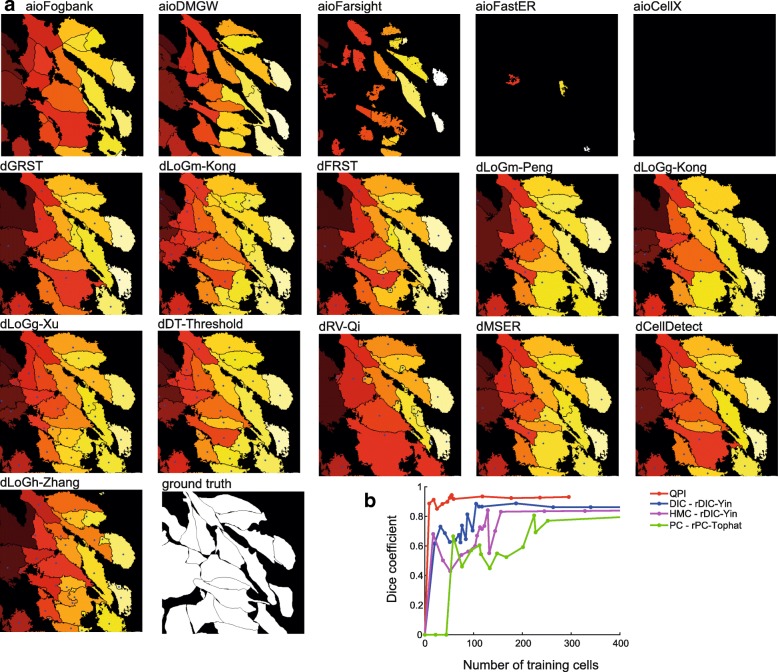
Seed-point extraction segmentation step and all-in-one segmentation approaches. **a** Results of segmentation, representative image of rDIC-Koos-reconstructed DIC image followed by foreground-background segmentation with Traniable Weka Segmentation. Blue points indicate seeds based on which cells are segmented using marker-controlled watershed. Note absence of seed-points for “all-in-one” segmentation approaches. **b** Dependency between number of cells used for training and Dice coefficient for Celldetect

Multiscale LoG filters (dLoGm-Peng and dLoGm-Kong) perform similarly as generalized versions (dLoGg-Kong and dLoGg-Xu), but Hessian-based LoG (dLoGh-Zhang) were significantly worse in some cases. As for the traditional microscopic methods, LoG approaches enables the highest achievable segmentation efficacy. It was found out that particular combinations of reconstruction-LoG filter perform better than others; an optimal reconstruction-seed-point extraction combination is rDIC-Koos followed by dLoGm-Peng for DIC, rDIC-Koos plus dLoGm-Kong for HMC, and rPC-Tophat plus dLoGm-Peng for PC. Moreover, there were dramatic differences in cell detection between QPI and the remaining contrast-enhancing microscopic methods. On the other hand, there were no differences with Dice coefficient 0.9 for both QPI and DIC with dLOGm-Kong (Fig. 4).

Hessian variant dLoGh-Zhang achieved low segmentation efficacy on our samples of adherent cells (of various sizes) due to the use of one estimated optimal kernel size only (see Table 2). dLoGg-Kong originally completely fails for some modalities due to the wrong cell size estimation caused by sub-cellular structures, which produce higher signal then cells. This was eliminated by introducing a new *σ*_*min*_ parameter, limiting the lower scale.

Regarding the computational times, LoG-based are among faster techniques, being surpassed only by the distance transform.

#### Radial symmetry transform-based strategies

Compared to the computationally-simple LoG-based techniques, the dFRST [31] and generalized dGRST [32] provide better results for unreconstructed QPI images and, notably, for unreconstructed HMC and PC images. On reconstructed data, a possible application is for PC data with results very close to QPI segmentation. Nevertheless, computational times in the orders of hundreds of seconds need to be taken into account.

#### Radial voting

Radial voting (dRV-Qi) approach [33] does not achieve the results of fast LoG-based strategies for all microscopic modalities, either raw or reconstructed, while being computationally comparable to radial symmetry transform-based approaches. Thus, it is considered not suitable for such data.

#### Distance transform

The strong advantage of the distance transform [15] is its speed, which is the highest among other seed-point extraction strategies. Segmentation efficacy of the tested version with optimal thresholding (dDT-Threshold) is the highest among all microscopies except for PC, but image reconstruction is needed. An alternative approach is to use WEKA for binary image generation (dDT-Weka), where cells are less separated than in a case of optimal threshold.

#### Maximally stable extremal region

Compared to the relatively consistent performance of LoG between microscopic techniques, the dMSER approach [34] is distinctly more suitable for HMC reconstructed by rDIC-Koos and PC reconstructed by rPC-Yin, where the segmentation performance as well as computational requirements are identical or similar to LoG.

#### CellDetect

The CellDetect approach uses [35] maximally stable extremal region for segmentation. Adherent cells in unreconstructed DIC/HMC/PC images are, however, dramatically heterogeneous structures. Thus, there are no elements registered for learning and thus the performance of CellDetect was similar to aioFastER methods. On the reconstructed data, it performs similarly as LoG- or distance transform-based methods. Nevertheless, because the trainable nature of this technique, enormous computational time demands must be taken into account (up to 100-fold higher than DT). Segmentation of microscopic elements of low shape heterogeneity (e.g. yeast) would profit from CellDetect significantly.

### Single cell (instance) segmentation

The data which underwent reconstruction, foreground segmentation and seed-point extraction were finally segmented by Marker-controlled watershed (MCWS) applied on distance transform or on images directly. Compared to previous steps, errors generated by this step have only minimal impact on overall segmentation quality, providing few-pixel-shifts to one or other adjacent cells. The distance transform approach is more universal but, in case the cells are well-separated, MCWS-only approach can provide better results. When compared to “all-in-one” segmentation strategies, the approach proposed by us provides dramatically better results except of proprietary software for quantitative phase imaging (see Table 2). With this in regard, the development of a new method which is strictly based on the nature of mass-distribution-QPI images could provide even better results.

**Fig. 5:**
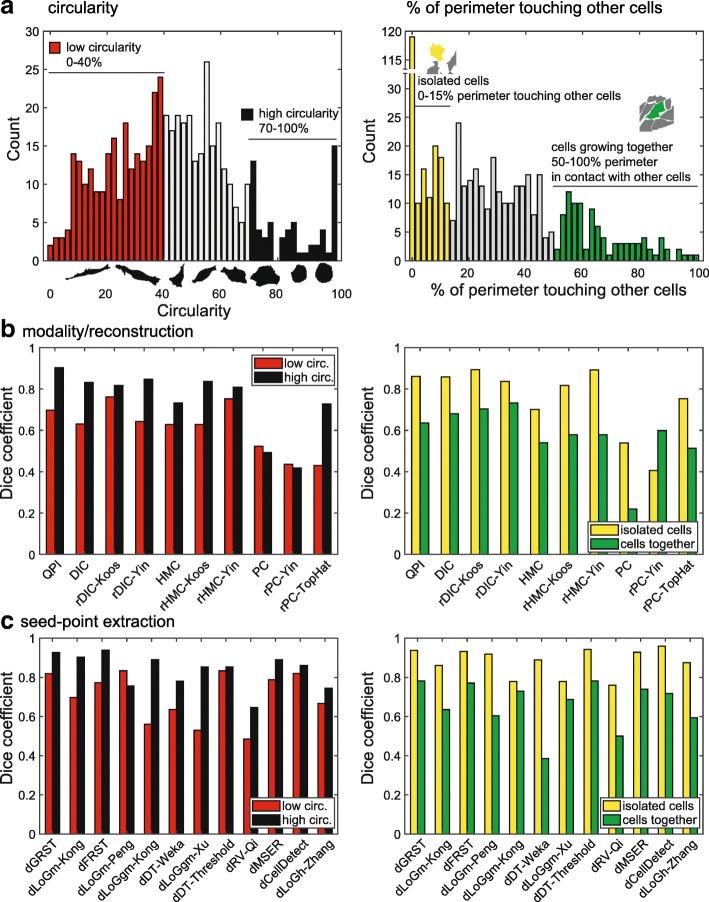
Cell segmentation efficacy and cell morphology. **a** histograms showing distribution of circularity and level of contact with other cells (shown as percentage of cell perimeter touching with other cells. Based on histograms, low/high circularity and isolated/growing together groups were created. **b** effect of cell reconstruction, on segmentation accuracy, subset of low/high circularity and low/high contact with other cells (for this step, dLoGm-Kong was used in next segmentation step for all methods). **c** effect of various Seed-point extraction methods, effect of low/high circularity and low/high contact on segmentation efficacy. Last step is shown for QPI data only

Finally, it was assessed how the segmentation accuracy’s individual steps are affected by morphological aspects of cells. Following aspects were studied (Fig. 5): cellular circularity and level of contact of cells with other cells (isolated cells vs cells growing together in densely populated areas, expressed as a percentage of cellular perimeter in contact with other cells). The circularity ranged 38.2 to 63.5%, median 51.2%, (percentage of cells with a circularity 100%: 2.1%), the percentage of perimeter ranged 4.1–41.9%, median 22.0% (percentage of cells with no contact with others 21.7%). Cells with circularity ranges 0–40% and 70–100% were considered low- and high-circularity cells. Regarding the degree of contact with other cells, cells whose 0–15% and 50–100% of perimeter was in contact with other cells were designated “isolated” and “growing together”, respectively.

It was found out that the reconstruction method does not affect a difference in segmentation accuracy between highly- and low-circular cells (the segmentation accuracy in highly circular cells is in average 15% better for all reconstruction methods) without significant variations for individual methods. Seed-point extraction, however, is much more cell-shape-dependent (Fig. 5c). Because these methods are blob detectors by nature, the result is better for more circular cells with most methods. However, the dDT-Treshold and dCellDetect are not affected by circularity and are among the most efficient segmenting tools at the same time.

Regarding the effect of a degree of contact with other cells, method of image reconstruction does not affect a difference in segmentation between densely and sparsely populated areas (20% better segmentation results for isolated cells). Seed-point extraction accuracy is however even more profoundly affected by a level of contacts with other cells (in average 25% better segmentation for isolated cells).

## Discussion

During the last two decades, the amount of approaches to segment microscopic images increased dramatically. The precise segmentation of label-free live-cell microscopic images remains challenging and not completely solved task. Furthermore, different microscopic techniques make this task more difficult due to different image properties provided.

Accordingly, the aim of this study was to compare the most heterogeneous spectrum of segmentation methods to real data of the same cells from multiple contrast microscopic modalities. The properties of each processing step has been evaluated and segmentation accuracy has been compared.

We used human adherent cells, which are much more heterogeneous in shape and thus much bigger challenge for segmentation than the segmentation of spherical bacteria or yeast. Based on the described results, we can now summarize, discuss and suggest several findings directed to both biologists and bioinformaticians from different points of view.

### Segmentability of microscopic techniques

When considering a microscopy technique for label-free segmentation, there were no dramatic differences in the segmentation efficacy between DIC, HMC or PC. However, the highest segmentation efficacy was obtained when QPI microscopy was used due to the higher image quality (without significant artefacts and high image contrast). In principle, approaches originally intended for fluorescence segmentation are applicable for these images. QPI technique should be also the choice, when a fast, high throughput segmentation is desirable, because no image reconstruction is needed and simple thresholding with MSER - Seeded watershed provides satisfactory results.

### Performance of segmentation steps

Regarding individual processing steps, the most crucial are image reconstruction and seed-point extraction methods. Foreground-background segmentation, on the other hand, can be considered the least problematic part, where no dramatic differences between methods were observed, except that learning-based approaches scored better. Regarding the seed-point extraction, however, a reconstructed image is needed for almost all approaches (except dDT-Weka), making seed-point extraction dependent on precise reconstruction. Not all foreground-background segmentation methods need reconstructed images, because some are compatible with raw DIC or PC images (e.g. sWeka, sLS-Caselles, sEGT) and generally perform well. Omitting the reconstruction step will need the seed point extraction methods applicable to raw data (eg. dDT-Weka or DT with different foreground-background segmentation), which can slightly reduce the quality of cell segmentation. It was also evident, that not all reconstruction algorithms are suitable for the seed-point extraction (high Dice coefficient in the foreground segmentation step does not guarantee suitability for the seed-point extraction). It also cannot be stated that the time-consuming methods are dramatically better-performing in the seed-point extraction. Here, the learning-based approach provided better results, too. Below we provide short workflow summary for each microscopic technique:

- QPI – this technique usually provides images with the best image properties with respect to automated image processing. We observed that Weka probability map segmented with Graph-cut, followed by seed-point extraction with dGRST and finally segmented by Marker-controlled watershed gives superior results. In general, any segmentation approach used, QPI gained the highest segmentation efficacy.
- PC – for this modality we suggest simple and fast reconstruction with Top-Hat filter, and dGRST or dFRST for seed-point extraction. Graph-cut applied to probability Weka probability maps produce best foreground-background segmentation. Final segmentation is slightly improved if Marker-controlled watershed is applied to distance transform image (instead of intensity image).
- DIC/HMC – the images from these modalities are similar, which leads us to suggestion that the same pipeline can be applied to both. We suggest to use rDIC-Koos method for reconstruction and Graph-cut applied to probability Weka probability maps for foreground-background segmentation. Thresholding with distance transform (dDT-Threshold) is best for seed-point extraction, finally segmented by Marker-controlled watershed. Although DIC and HMC have a lot of similar features, DIC produce generally better results.

All-in-one packages are extremely popular in biologist community and more or less provide the complex solution for single cell segmentation task. However, these packages implement common image processing methods (some of them described here) and together with graphical user interface and interactions, provide rich possibilities for segmenting the images. We can conclude that FogBank and CellProfiler tools achieve the highest segmentation efficacy among these approaches (without need of programming skills) and it is also universal for various imaging modalities. Both FogBank and CellProfiler use a similar generalizable approach based on the combination of watershed and distance transform, however, CellProfiler also includes a possibility to build complete cell analysis pipelines and as such should be default choice without programming.

### Deep-learning remarks

Intentionally, our focus was set on a spectrum of traditional strategies while the rapidly-developing spectrum of deep-learning-based segmentation was omitted. The main practical limitation of application of deployment of pre-trained U-net or other deep learning method (transfer learning) is the need for sufficiently large training dataset (covering different modalities and cell types/shapes). However, the image databases for segmentation tasks are not as large and complex as ImageNet [36], which became a standard for pre-training of classification-based networks. For this reason, available models use only pre-trained encoder [37], which is pre-trained for classification on ImageNet. As such, we leave this investigation for future work, where deeper-comparison is highly needed, especially for different amounts of training data and from view of computational requirements.

Despite the tremendous success of deep learning approaches applied in many computer vision tasks including live cell imaging, there is no straightforward way how to use these methods for cell segmentation of touching dense populated cells. One of the approaches to achieve separated mask for each cell is to predict simple binary foreground mask, but giving higher weight to correct prediction on the boundary as in [38]. Another simple solution is to predict three pixel classes – background, foreground and cell boundary as in [39], which provides better separation of cells. On the other hand, deep learning can be also used for cell detection by regression of 2D Gaussians on the position of centroids as in [40]. In [41], authors combined detection and segmentation into simultaneous prediction with one U-Net network, where one prediction map predicts distance to cell boundary (after thresholding we obtain foreground-background segmentation) and second map predicts distance to cell centroid (detections are obtained with local maxima detection). These techniques are very promising, however, their testing is out of scope of this paper because our dataset is not sufficiently large for training of these algorithms and there is no standard way how to use deep learning for cell segmentation, leading to enormous number of possible setups to test in order to achieve fair comparison to classical methods.

### Remarks and limitations

Because of Matlab platform was used, the information regarding computational time is approximate with a large software-dependent space for its reduction. All segmentation steps were performed in a sequential way. Thus parallel processing may provide a distinct improvement for most of the methods, but this was beyond the scope of this study. Based on a distinctive difference in a segmentation accuracy between “all-in-one” methods and individual methods reviewed herein, well-performing methods usually have more than three parameters to be set (usually not even corresponding with morphological features of the cells). Thus it is still difficult, if not impossible, to automatize the whole segmentation process. In a spite of this, deep-learning approaches provide some alternative – instead of setting optimized parameters, user needs just to provide a training dataset.

Although there are several excellent reviews on such segmentation, a study practically comparing the to-date best-performing approaches on real data from various microscopic techniques is still missing. In [42] the authors review a broad spectrum of segmentation methods to segment histological images. In [43] the authors focus on available tools with GUI. The author of [44] summarizes historical progress of cell segmentation methods. There are also works on comparing QPI, DIC and PC, but in [45], the authors compare modalities without segmentation and in [46] authors test algorithms only on QPI data, without considering image reconstructions. In Ulman et al. [47] the authors compared segmentation and tracking on various microscopic methods, including 2D fluorescent, DIC and PC. Many detection errors can be eliminated with tracking. Thus the comparisons with our review might be rather relative. Similarly to our results, one of the best-performing algorithms “KTH-SE” used a relatively simple thresholding together with a precise seed-point extraction (tracking in their case). This underlines the fact that a precise seed-point extraction is the most crucial segmentation aspect. Also a segmentation performance was significantly lower in the “Fluo-C2DL-MSC” dataset characteristic by low circularity of cells.

Our study has several limitations. These include the focus on the segmentation of adherent cells, not those cultivated in the 3D matrix or suspension-cultured counterparts. Also the ground truth manual segmentation was performed by a human, although experienced biologist. The problem of overlapping cells was present, although relatively rare. Using the learning-based approaches it was demonstrated that those surpass the transitional strategies. This predicts a future success for deep-learning methods and probably also their future superiority. Also, in accordance with cell time-lapse trend in microscopy, cell segmentation is just the first part of the story with cell tracking being another one.

## Conclusion

In this study, we performed a comprehensive testing of image processing steps for single cell segmentation applicable for label-free images. We searched for published methods, which are used by biologists and bioinformaticians, we assessed the suitability of used data and we carefully tested particular segmentation steps (image reconstruction, foreground-background segmentation, seed-point extraction and cell segmentation) and compared them with available “all-in-one” approaches. As expected, learning-based methods score among the best-performing methods, but well-optimized traditional methods can even surpass these approaches in a fraction of the time. We demonstrated that the image reconstruction step makes it possible to use segmentation methods not directly applicable on the raw microscopic image.

Herein we collected a unique set of similar field-of-view image of the same cells acquired by multiple microscopic techniques and annotated by experienced biologist. The raw and reconstructed data is provided, together with the annotated ground truth and Matlab codes of all approaches.

## Methods

### Dataset

#### Cell culture and culture condition

PNT1A human cell line was used in the experiment. This cell line was derived from normal adult prostatic epithelial cells immortalized by transfection with a plasmid containing SV40 genome with defective replication origin. The cell line was purchased from HPA Culture Collections (Salisbury, UK). PNT1A cells were cultured in RPMI-1640 medium supplemented with antibiotics (penicillin 100 U/ml and streptomycin 0.1 mg/ml) with 10% fetal bovine serum (FBS). Prior microscopy acquisition, cells were maintained at 37^∘^C in a humidified (60%) incubator with 5*%*C*O*_2_ (Sanyo, Japan). Intentionally, high passage number of cells was used (>30) in order to describe distinct morphological heterogeneity of cells (rounded and spindle-shaped, relatively small to large polyploid cells). For acquisition purposes, cells were cultivated in Flow chambers *μ*-Slide I Luer Family (Ibidi, Martinsried, Germany).

#### Microscopic image acquisition and dataset characteristics

QPI microscopy was performed on Tescan Q-PHASE (Tescan, Brno, Czech Republic), with objective Nikon CFI Plan Fluor 10×/0.30 captured by Ximea MR4021MC (Ximea, Münster, Germany). Imaging is based on the original concept of coherence-controlled holographic microscope [48, 49], images are shown as a 32bit file with values corresponding to pg/ *μ*m^2^ recalculated from radians according to Barer and Davies [50, 51].

DIC microscopy was performed on Nikon A1R microscope (Nikon, Tokyo, Japan) with a Nikon CFI Plan Apo VC 20×/0.75 objective captured by a Jenoptik ProgRes MF CCD camera (Jenoptik, Jena, Germany).

HMC microscopy was performed on Olympus IX71 microscope (Olympus, Tokyo, Japan) with Olympus CplanFL N 10×/0.3 RC1 objective captured by Hamamatsu Photonics ORCA-R2 CCD camera (Hamamatsu Photonics K.K., Hamamatsu, Japan).

PC microscopy was performed on a Nikon Eclipse TS100-F microscope, with a Nikon CFI Achro ADL 10×/0.25 objective captured by Jenoptik ProgRes MF CCD camera.

The captured dataset characteristics are summarized in Table 3. All data were manually segmented by an expert in cell biology as ground truth for segmentation and detection. Same areas of sample were captured using these microscopes, but due to the cell movement and different FOV size the overlap is not absolute.

**Table 3:** Data-set summary

Modality	FOV size	Image size	Num. of FOVs	Num. of cells
QPI	376 ×376 *μ*m	600 ×600 px	18	637
PC	1253 ×944 *μ*m	1360 ×1024 px	10	2387
DIC	627 ×472 *μ*m	1360 ×1024 px	11	862
HMC	867 ×660 *μ*m	1344 ×1024 px	11	1297

### All-in-one segmentation tools

Here are described “all-in-one” approaches (designated with “aio” prefix).

#### aioFARSIGHT

FARSIGHT toolkit 0.4.5 module Nucleus editor [2] consists of an automatic Poisson threshold binarization refined with graph-cut (applied on a binary foreground image) and produces initial segmentation containing cell clusters. Next, Multiscale Laplacian-of-Gaussian is used to produce feature map (image where blobs are enhanced - see “LoG filters” section for more details), which is segmented by local clustering algorithm. This clustering algorithm then produces rough cell cluster separation. Finally *α*-Expansions (multilabel graph cut) is used to refine segmentation, with novel method of Graph colouring for more efficient computation (see [2] for more details).

The first set of parameters was cell-shape-derived: “min scale” and “max scale” (the minimum and maximum scale of the multiscale Laplacian of Gaussian filter) were set based on a measured radius of equivalent circle of cells, “xy clustering res” (resolution of the local maximum clustering) was set similarly as “min scale”, and “min object size” was set as the area of the smallest cell. The second set of parameters was optimized: “high sensitivity” (enable/disable high sensitivity binarization), “finalize segmentation” (enable/disable the segmentation refinement step), “use distance map” (enable/disable the use of the distance constraint to select the LoG scales), and “refinement range” (parameter sets the maximum distance that an initial contour can be shifted).

#### aioCellX

Dimopoulos et al. [3] approach consists of seed generation with gradient-based Hough transform, construction of membrane patterns images for each seeed (cross-correlation with estimated membrane profile) and segmentation of each such image with graph-cut. After that, statistical morphological outliers are removed and individual regions are combined (almost identical regions are merged and overlaps are resolved).

CellX includes a GUI, where user can interactively set cell size range, maximal cell length and estimated membrane profiles.

#### aioFogbank

In Chalfoun et al. [4] Fogbank, foreground is segmented with EGT. Seeds are detected as connected regions after percentile thresholding (with some distance and size constraints). Pixels above a defined percentile level are then connected to the nearest seed-point. Either intensity or gradient image and either Euclidean or geodesic distance are used for computation.

#### aioFastER

Hilsenbeck et al. [5] FastER applies MSER to construct component tree and SVM for classification of regions into groups of cells or false detections. Finally non-overlapping regions with the highest score are selected. It shares CellDetect similarities (see “CellDetect” section), but this algorithm uses 9 features for SVM classification only and does not compute globally optimal solution, thus being computationally faster. To achieve complete segmentation (not only detection as CellDetect), authors modified their approach on the algorithmic level. Size constraints of cells (measured min/max cell size) were set and “de-noise” parameter setting were optimized (off/on/strong). Larger number of FOVs used for training were tested without improvement.

#### Dry mass-guided watershed

The dry mass-guided watershed method (designated as aioDMGW) is a thresholding-based approach, implemented as a part of Analyzer module of Q-PHASE software 6.803 (Tescan, Brno, Czech Republic). First the phase image is slightly smoothed and foreground is separated from background using thresholding. Then watershed starting from the local maxima is performed. The decision of merging of touching segments, or leaving them separated, is based upon the sums of pixel values (i.e. dry mass) in each touching segment. The optimized parameters are: threshold; min segment sum (the minimum accepted sum of pixel values in each segment used to filter out noise and cell debris); max merge sum (the threshold of sum of pixel values of touching segments used to decide if the segments should be merged or left separated).

#### aioCellProfiler

The CellProfiler [10] is a strong segmentation tool, however, we perceive it more as a platform where a substantial part of the segmentation strategies used here can be reproduced. Nevertheless, we evaluated output of “IndetifyPrimaryObject” module, which combines thresholding and watershed. Watershed is used twice, for seed-point extraction and final single cell segmentation, and it is applied to either intensity or DT image. Additionally, module uses some smoothing and it remove seed-points bellow some allowed distance. Measured range of cell radiuses and optimal threshold (see Additional file 1) were used and we optimized betwen application to intensity or DT image for both steps.

#### Other all-in-one tools

Following algorithms were reviewed but not used in comparison with reasons stated below:

CellTracer [6] consists of 3 steps – foreground segmentation, border segmentation and cell segmentation by model fitting. This approach is more suitable for yeast- or bacteria-shaped objects (coccus- or bacillus-shaped with distinctive borders). Similar issues were observed in SuperSegger [7], CellSerpent [8] or CellStar [9].

### Image reconstruction techniques

DIC, HMC and PC image formation process can be described as convolution between the original image of the scene and 2D PSF. For PC images *PSF* is [13]

1 $$  {PSF}_{PC}(x,y) = \delta(x,y) - airy\left(\sqrt{x^{2}+y^{2}}\right)  $$

where *δ*(·) is Dirac delta function and *airy*(·) is Airy pattern. This leads to halo and shade-off artifacts (see Fig. 2). For DIC image *PSF* is difference of two Gaussians [52]:

2 $$  {} {PSF}_{DIC}(x,y) \,=\, -x u \exp\left(-\frac{x^{2}+y2}{\sigma}\right) - y v \exp\left(-\frac{x^{2}+y2}{\sigma}\right)  $$

where *σ* is Gaussian standard deviation and **u**=[*u* *v*]^*T*^ is unit vector specifying shear direction. It means that DIC image is derivation under shear direction visible as 3D-like topographical appearance (see Fig. 2). The inverse PSF then can be used for image reconstruction. The goal of these reconstruction algorithms is to produce image of blob-like cells qualitatively corresponding to cell mass (similar to QPI). The methods described bellow are designated with prefix “r” (reconstruction), original modality and author, where possible.

DIC reconstruction methods were well reviewed in [12]. Based on the results of this study, two methods were chosen: (a) fast, computationally-efficient Yin et al. approach [13] (in following parts designated as “rDIC-Yin”) and (b) more computationally-demanding Koos et al. [12] (designated as “rDIC-Koos”). HMC images have the similar properties as DIC and therefore the same reconstruction algorithms were tested.

For PC reconstruction [14], two methods were chosen (a) more complex computationally-demanding method based on PSF model (designed as “rPC-Yin”) (b) simple Top-hat filtering (designated as “rPC-Tophat”).

#### rDIC-Koos

Method proposed by Koos [12] (rDIC-Koos) uses an energy minimization with data term and total variation regularization term

3 $$ E=\frac{1}{2} \iint_{\Omega} (\mathbf{u} \cdot \bigtriangledown (K \ast \hat{f})- \mathbf{g})^{2} + w_{s} |\bigtriangledown \hat{f}| d\Omega  $$

where · denotes dot product, ▽ denotes gradient, **u**=[*u* *v*]^*T*^ is unit vector specifying shear direction, *Ω* is image domain and *K* is kernel which approximate PSF without derivative (Gaussian function), where ▽*K*=*PSF*_*DIC*_(*x*,*y*). Euler-Lagrange equation of data term for symmetric kernel *K* leads to

4 $$ u \partial_{x} g + v \partial_{y} g - \iint_{W} K \left(u^{2} \partial_{x}^{2} \hat{f}+ 2 u v \partial_{x} \partial_{y}+v^{2} \partial_{y}^{2} \hat{f}\right)=0  $$

where *∂*_*x*_ and *∂*_*y*_ denotes partial derivatives and *W* is a local window (with size of kernel). Finally, this can be solved with gradient descent iterative method as

5 $$ {{} \begin{aligned}  \hat{f}^{(t+1)}&=\hat{f}^{(t)} - w_{a} \left(u^{2} \partial_{x}^{2} \hat{f}^{(t)}+ 2 u v \partial_{x} \partial_{y} \hat{f}^{(t)} + v^{2} \partial_{y}^{2} \hat{f}^{(t)}\right) \ast K \\&+ u \partial_{x} G + v \partial_{y} G - div\left(\frac{\bigtriangledown \hat{f}^{(t)}}{||\bigtriangledown \hat{f}^{(t)}||}\right) \end{aligned}}  $$

where $\hat {f}^{(t+1)}$ is reconstructed image in next iteration, *div* denotes divergence. Last term is proposed by total variation regularization.

Besides of shear angle, which is assumed to be known (or recognizable from image - typically multiple of 45), rDIC-Koos method has three parameters - weight of smoothness (total variation) regularization *w*_*s*_, step size of gradient descent *w*_*a*_ and number of iteration *it*. Smooth regularization sets compromise between noise elimination and details preservation. Too large step size leads to method divergence and too small step size leads to slow convergence. Number of iterations has a small influence on the result; default value 20000 was used. For setting of other parameters see Additional file 1.

#### rDIC-Yin

Yin et al. [13] presented a reconstruction method for DIC images (rDIC-Yin) working with multiple shear directions, but with some simplification in equations it also works on images with one shear angle direction. Authors assumed that distortion of the microscope can be modeled by convolution with PSF

6 $$ \mathbf{g}=\mathbf{d} \ast \mathbf{f}  $$

where **d** is PSF (in general a directional first-derivative-of-Gaussian kernel, but simple difference without Gaussian is used for simplification), **g** is acquired image and **f** is original image. Simple inverse filtering leads to highly noisy images, which can be reduced by regularization. This can be achieved with optimization of energy function which must be minimized over whole image domain

7 $$ \mathbf{E}(\hat{\mathbf{f}})=(\mathbf{d} \ast \hat{\mathbf{f}} - \mathbf{g})^{2} + w_{s}(\mathbf{a} \ast \hat{\mathbf{f}})^{2} + w_{r} \hat{\mathbf{f}}^{2}  $$

This equation is composed of data term, smooth term and sparse term (all with *l*_2_ penalization, where *w*_*s*_ and *w*_*r*_ are weights for the smooth and sparse regularizations, respectively). $\hat {\mathbf {f}}$ is reconstructed image (approximation of **f**). Smoothness is achieved by setting a restored pixel value to be close to the average of its neighbors (where **a**=[1,1,1;1,−8,1;1,1,1]/8). Sparse regularization causes the value of background pixels to be close to zero. Optimization of function has close-form solution in Fourier space ($\hat {\mathbf {F}}=\mathcal {F}\{\hat {\mathbf {f}}\}$ etc.)

8 $$ \hat{\mathbf{F}}=-(\mathbf{D} \odot \mathbf{G}) \oslash (w_{s} \mathbf{A}\odot\mathbf{A} +w_{r}-\mathbf{D}\odot\mathbf{D})  $$

where “ ⊘” and “ ⊙” denotes element-wise division and multiplication, respectively.

Besides shear angle, rDIC-Yin has two parameters only, *w*_*s*_ and *w*_*r*_, which set smoothness and sparse regularizations, respectively.

#### rPC-Yin

In [14] Yin et al. used a deconvolution with sparse constraint regularization to reconstruct PC images. This method was further expanded with dictionary of diffraction patterns [53], which deals with problematic mitotic cells. This method is in fact a segmentation method as presented in the Su at al. paper [53] and it therefore cannot be used as preprocessing (i.e. reconstruction) step. rPC-Yin [14] is very similar to rDIC-Yin [13] with modified equation 7 to linear equation system with *l*_1_ penalization for the sparse term.

9 $$ \mathbf{E}(\overline{\hat{\mathbf{f}}})=(\mathbf{H}\overline{\hat{\mathbf{f}}} - \overline{\mathbf{g}})^{2} + w_{s}\overline{\hat{\mathbf{f}}}^{T}\mathbf{L} \overline{\hat{\mathbf{f}}} + w_{r} |\Lambda \overline{\hat{\mathbf{f}}}|  $$

where $\overline {\hat {\mathbf {f}}}$ and $\overline {\mathbf {g}}$ are vectorized restored and acquired images, **H** is the transfer matrix of the image formation model and **L** is Laplacian matrix (corresponding to different expression of operators **d** and **a** in the equation 7). *Λ* is positive diagonal matrix defining sparseness, *w*_*s*_ and *w*_*r*_ are weights for the smooth and sparse regularizations. Because of *l*_1_ penalization of sparseness (known to be better than *l*_2_) there in no closed-form solution. It can be solved with an iterative algorithm which is based on non-negative multiplicative updating (for more implementation details see [14]). PSF (which leads to **H**) is then modeled by the equation 1, where airy pattern is

10 $$ airy(r)=R \frac{J_{1}(2 \pi R r)}{r} - (R-W) \frac{J_{1}(2 \pi (R-W) r)}{r}  $$

where *R* and *W* are PSF-dependent parameters - outer radius (*R*) and ring width (*W*) of phase ring and *J*_1_(·) is the first order Bessel function of the first kind. rPC-Yin has also optimization parameters *w*_*s*_ and *w*_*r*_ which define weights of components of optimized energy function. Other parameters not discussed in [14] were set to default value (*radius*=2,*epsilon*=100,*gamma*=3,*scale*=1,*maxiter*=100,*tol*=10^−16^). Because of large computational time, optimization of PSF and optimization parameters was done separately - first proper PSF was found (other parameters set to default value *w*_*s*_=1 and *w*_*r*_=0.5) and then optimal *W* and *R* values were used in optimization of *w*_*s*_ and *w*_*r*_.

#### rPC-Tophat

Top-hat filtering (referred here as rPC-Tophat) was used by Thirusittampalam et al. [15] and Dewan et al. [16] for halo artifacts elimination. This simple heuristic approach shows very promising results and it is considered as the next PC reconstruction technique in this paper.

Reconstruction based on top-hat filtering with disk-shaped structuring element has only one adjustable parameter - radius of structuring element, which is roughly equal to the radius of the cell, with optimal value *r*=16.

### Foreground-background segmentation

Thirteen methods has been tested and to make it more clear, the methods are designated with prefix “s” (segmentation), original modality and the author, where possible.

#### Thresholding

Three threshold-based techniques were used for the foreground-background segmentation. Simple threshold (named as sST) and two automatic threshold algorithms, Otsu [17] (sOtsu) and Poisson distribution [2] (sPT).

Automatically determined thresholds varies between FOVs, so a better result can be expected. sOtsu assumes that gray-scale values are mixture of two Gaussian distributions. Nevertheless, for the adherent cell images the mixture of two Poisson distributions is sometimes more suitable [2], thus sPT was tested. For ST, threshold value was optimized with 100 steps between minimal and maximal value.

#### Empirical gradient threshold

Chalfoun et al. [18] described an empirical gradient threshold method (referred here as sEGT), which uses empirically derived model for threshold estimation. sEGT was described to work with different microscopic modalities (PC, DIC, brightfield and fluorescence) and is applicable also on the others, including raw (unreconstructed) images. sEGT utilizes a Sobel operator to compute absolute value of gradient, then the percentile-based threshold is found, followed by the binary morphological operations. Three parameters must be set beforehand: minimal cell size (removing small objects), minimal hole (removing small holes) and manual fine-tune (decreasing or increasing the estimated threshold). For all these methods minimal object size was determined from a ground true mask of the training images.

#### sPC-Juneau

Juneau et al. [19] described simple segmentation method (referred here as sPC-Juneau) designed for PC images. It computes a range map (difference between minimum and maximum in local window), which is then thresholded. Consequently, all holes and small objects in the binary image are removed. Thus these parameters are optimized: window size, threshold and minimal object size. Although originally designed for PC images, it is applicable for other modalities as well.

#### sPC-Phantast

Jaccard et al. [21] developed a software toolbox PHANTAST consisting of foreground segmentation techniques specialized for PC microscopy images. It computes local contrast

11 $$ C = \frac{\sqrt{G\ast I^{2} - (G\ast I)^{2}}}{G\ast I}  $$

where G is a Gaussian kernel with standard deviation *σ*. The resulting local-contrast image is then globally thresholded and halos are corrected. For halos correction, the gradient direction is computed by eight Kirsch filters (8 directions). Halo pixels are initialized with boundary pixels of binary image, then iteratively each halo pixel points to its gradient direction and two adjacent directions, where each of these three pixels is marked as halo if it is considered foreground (for bright halos gradient points in and for dark cells gradient points out). Maximum cell area fraction removed as halo is restricted and after elimination of halos, small objects and holes are removed. This leads to 5 parameters - Gaussian *σ*, threshold, halo area fraction, minimal hole size and minimal object size.

#### sPC-Topman

Topman et al. [20] described another method for foreground segmentation originally intended for PC images. This approach applies two filters, one with a small and one with a large local window computing the standard deviation, where both are thresholded. The result is an intersection of these two binary images, where binary image from large window is morphologically eroded (with morphological element of half the size of the large window) and final image is morphologically opened and closed. This leads to 4 parameters - two window sizes, threshold, and morphological element size.

#### LevelSets

Matlab implementation of level-set method with function *activecontour* was used. This implementation includes an edge-based method [22] (referred as sLS-Caselles) and region-based method [23] (referred as sLS-ChanVese). Both methods use a Sparse-Field implementation [54] for contour evolution and both have two adjustable parameters - smoothness of the result contour and additional force, which leads to a tendency of the contour to grow or shrink. While sLS-ChanVese segments the image into two regions based on the mean region intensities, sLS-Caselles segments the image based on the edges. The level-set methods were initialized with morphologically-dilated binary results of Weka segmentation, because it provides similar initial contours for all modalities. Number of iterations of the evolution was set to 1000, which was shown to be enough for all types of images and all parameter settings.

#### Trainable Weka Segmentation

Next, a machine learning tool for microscopy pixel classification Trainable Weka Segmentation v.3.2.13 was used [26] (designated as sWeka). Compared to previous foreground-background segmentation strategies, this approach was primarily used directly on the raw data. Weka was trained using the following default training features (Gaussian blur, Sobel filter, Hessian eigenvalues, difference of Gaussians filter, membrane projections) as well all remaining available filters (variance filter, minimum filter, maximum filter, median filter, anisotropic diffusion, bilateral filter, lipschitz filter, kuwahara filter, gabor filters, Sobel filter, laplacian filter, structure, entropy filter). For these filters it is also possible to set a *σ* range, which specifies the filter size. Other parameters were set to default values, random forest classifier was set to 200 trees (WEKA FastRandomForest). Because of learning nature of this approach, the effect of following factors on segmentation efficacy was optimized: (a) number of fields of view used for learning (b) training features used for learning (“all” and “default” training features), (c) effect of various fields of view used for training (one continuous area in one FOV, or smaller patches of same sizes from multiple FOVs), (d) size of FOV used for learning (increasing the area from 6×6 px to 1360×1024 px). Moreover, probability maps were exported and used for further analyses.

#### Ilastik

Another tested machine learning tool for pixel classification was Ilastik v.1.3.0 [25]. Ilastik uses a random forest classifier [55] with 100 trees and is very similar to WEKA. Ilastik was launched using the following settings: enabled all training features: raw intensity, gradient magnitude, difference of Gaussians, Laplacian of Gaussian, structure tensor eigenvalues and the Hessian matrix eigenvalues - all with 7 Gaussian smoothings with *σ*=0.3−10*px*.

Ilastik was optimized accordingly as Weka. It allows a computationally expensive automatic selection of suitable features. Based on a first optimization step, there was no significant difference between “optimal” and “all” features. Thus, in a spite of this and a fact that Ilastik has less available features then WEKA, “all” features were used in further steps.

#### Graph-cut approach

An ImageJ plugin for Graph-Cut (v. 1.0.2) based on the reimplementation of Kolmogorov’s maxflow v3.01 library [24] was used. The following data were used as an input for Graph-Cut: (a) Probability maps generated by Weka (referred as sWekaGraphCut), (b) images reconstructed with approaches described in “Image reconstruction approaches” and (c) raw image data (both referred as sGraphCut). There are two parameters to be optimized: terminal weight and edge weight. Edge weight (designated as “smoothness” in the GUI, range 0-10) reflects a penalty for label changes in the segmentation (higher values cause smoother result). Terminal weights (designated as “foreground bias”, range 0-1) correspond to a cost of assigning background pixels to the foreground.

Terminal weights (foreground bias in GUI) affect the segmentation efficacy distinctly, thus its optimization is crucial. On the other hand, edge weight (smoothness) corresponds to the size of individual cells and has been roughly estimated from 0.4 to 0.8 for used cell sizes (between 1000 and 4000 pixels, respectively).

### Cell detection (seed-point extraction)

The cell detection (seed-point extraction) plays a key role in the segmentation of the overlapping objects. For densely clustered and touching cells a precise cell detection has the most significant influence to the final segmentation accuracy. The primary goal in the cell detection is to recognize the presence of the individual objects in the image. Finally, combination of successful foreground-background separation followed by identification of individual cells enable to segment individual cells. There is a considerable amount of methods for cell detection and the mostly used and cited methods are described and evaluated in this paper. Because most of the described methods require blob-like cells, image reconstruction is necessary in most cases (except LoG and generalized LoG filters by Kong et al. [28]).

The tested seed-point extraction methods usually include parameters related to the cell radius (minimal and maximal). For this reason we estimated these values from the ground truth masks. Background segmentation from the previous step was used to eliminate falsely detected seeds in the background. Some of the tested methods already include this step (e.g. dLoGg-Xu [29]). The binary background masks produced by trainable Weka segmentation were used for this purpose. For clarity, the methods described bellow are designated with prefix “d” (detection), image processing approach and author, where possible.

#### LoG filters

Because of distinctive popularity of the LoG filter for the blob object detection, many modifications of this detector exist, e.g. multi-scale LoG, Hessian LoG, generalized LoG. LoG filter at a scale *σ* is defined by equation

12 $$ LoG(\mathbf{x},\sigma)= \nabla^{2}G\left(\mathbf{x},\sigma\right)=\frac{\sigma^{2}-||\mathbf{x}||^{2}}{2\pi\sigma^{6}}e^{\frac{-||\mathbf{x}||^{2}}{2\sigma^{2}}},  $$

where *G* is 2D Gaussian function, **x**=(*x*,*y*) and ||·|| is Euclidean norm [27]. In principle, this filter works as a matched filter for blobs.

Multi-scale LoG filtering uses a bag of LoG filters with *m* different sigma values, which leads to *m*−*D* feature space. As proved by Lindeberg [56], LoG responses must be normalized *LoG*(**x**,*σ*)_*norm*_=*σ*^*γ*^*LoG*(**x**,*σ*) for scale invariance, where *γ*=2 for scale invariance, but it can be refined for a preference of larger or smaller objects.

Peng et al. [27] used Maximum Intensity Projection (MIP) of the series of LoG-filtered images $MIP(\mathbf {x})=\max \limits _{\sigma }({LoG}_{norm}(\mathbf {x},\sigma))$, with threshold applied to resulting 2D image, where binary objects correspond to the detected cells. This method (further designated as dLoGm-Peng) has the following parameters: minimal sigma *σ*_*min*_, maximal sigma *σ*_*max*_, sigma step *Δσ*, *γ* and threshold.

Kong et al. [28] searched for local maxima higher than defined threshold in whole *m*−*D* LoG scale space with elimination of overlapping regions by a pruning process. In the pruning process, the overlapping blobs are eliminated, where only blob with larger value in *m*−*D* scale space is preserved. This method has these parameters: *σ*_*max*_, sigma step *Δσ*, *γ*, threshold and maximal overlap ratio. Here for *σ* the logarithmic step is used. This method is referred as dLoGm-Kong.

Hessian analysis of LoG (referred as dLoGh-Zhang) described by Zhang et al. [30] uses the same bag of LoG-filtered images, but optimal scale identification and cell center detection is different. It is known, that local Hessian matrix is positive definite for blob-like structures. The Hessian *H* (computed from LoG-filtered image) at position (x,y) can be approximated with differences in 2×2 neighborhood. Each connected region with a positive definite Hessian is considered as cell, where *H* is a positive definite matrix when *H*_11_ is positive and *det*(*H*) is positive.

13 $$ H(x,y,\sigma)= \left(\begin{array}{cc} \frac{\partial LoG(x,y;\sigma)}{\partial x^{2}} & \frac{\partial LoG(x,y;\sigma)}{\partial x \partial y} \\ \frac{\partial LoG(x,y;\sigma)}{\partial y \partial x} & \frac{\partial LoG(x,y;\sigma)}{\partial y^{2}} \end{array} \right).  $$

Optimal is considered a such scale where the mean intensity of the LoG-filtered image is maximal on the positive definite locations, and these positive definite regions are the detected cells. Method is insensitive to choice of range and steps of *σ*, which leaves only *γ* parameter to be optimized. Zhang [30] also uses unsupervised classification to identified true cells only, but in our case this leads to deterioration of the results only and thus was not included in the testing.

Intuitively rotationally-symmetric LoG kernels are very sensitive to irregular cell shape. For this reason Kong et al. [28] proposed a generalized LoG filter (referred as dLoGg-Kong) for the detection of the elliptical shapes. They derived an equation for elliptical kernel with two standard deviations *σ*_*x*_, *σ*_*y*_ and orientation *Θ*. Method also includes a specific scale normalization with a parameter *α* and automatic choice of sigma range based on the initial analysis with circular LoG filters. For every pixel position, a feature image is created as a sum of all filter responses and detected cells are local maxima in this image (see [28] for more details). Thanks to the automatic *σ* estimation, there is one parameter only - *α*. Method uses integer kernel sizes smaller than estimated *σ*_*max*_. Small kernels produce false peaks on a sub-cellular structures in our data. These artefacts are eliminated by adding a *σ*_*min*_ parameter, which corresponds to a minimal cell radius.

Xu et al. (referred as dLoGg-Xu) [29] sped up this technique by summation of the filters with the same kernel orientation *Θ*, which is possible thanks to the distributive property of convolution. Instead of automatic estimation of *σ* range, they estimate it from cell radii. Moreover this technique includes a different normalization (without parameter) and mean-shift clustering for elimination of multiple-time detected seeds. Parameters of this method are: *σ* range and mean shift window size.

A similar approach was described also in Peng et al. [27] method. Parameter range of *σ* is estimated based on cell radius as $\sigma =r/\sqrt {2}$. For dLoGm-Peng we used estimated *σ*_*max*_ and *σ*_*min*_. Step of *σ* (*Δσ*) is insensitive parameter, therefore we set it to 1. For setting of other parameters see Additional file 1. Authors [27] used *γ*=2, which is proven to lead to the theoretical scale invariance. When *γ*<2 the smaller objects are preferred, for *γ*>2 the larger objects are preferred. Appropriate setting of *γ* leads to mean Dice coefficient improvement +0.089 for dLoGm-Peng method and for this reason we add *γ* to optimized parameters for both dLoGm-Peng and dLoGm-Kong methods. Similarly for dLoGm-Kong we used estimated *σ*_*max*_ and *σ*_*min*_ with 13 logarithmic steps like the authors[28] (for other parameter settings see Additional file 1). Extension by *γ* parameter leads to 3 parameters (besides of cell radii), which are sensitive and must be properly set. Both generalized LoG methods try to avoid parameters setting, where dLoGg-Xu has cell size-related parameters only (we set it based on estimated radius) and dLoGg-Kong has one adjustable parameter - scale normalization factor, but cell size estimation is automatic. Both generalized LoG methods are computationally expensive (see Table 1), but dLoGg-Xu reduces the computational time by a reduction of number of convolutions.

#### Distance transform

Distance transform (DT) of foreground image is defined as a distance to the nearest background pixel (Euclidean distance is chosen as metric). Local maxima of the generated distance map are considered as cells. This method often detects many false cells. For this purpose h-maxima transform is used [15], which uses a grayscale morphology for elimination of small local maxima, where parameter *h* sets the depth of local maxima to be eliminated. We used two modifications of this method; dDT-Threshold, where binary foreground is computed with optimized threshold and dDT-Weka, where foreground from Weka segmentation is used. Other parameter of this method is maximal size of objects and holes, which are eliminated before applying of the DT.

#### Fast radial-symmetry transform

Fast radial-symmetry transform [31] (referred as dFRST) is a general method for the detection of circular points of interest applicable to approximately circular objects. Pixels with absolute value of gradient greater than threshold *β* vote in its gradient direction at the distance of radius *r*∈*R*, where *R* is set of radii, determined based on object/cell size. If bright blobs are only considered detection, positions of affected pixel is given by an equation

14 $$ P(\mathbf{x})=\mathbf{x}+round\left(\frac{g(\mathbf{x})}{\lVert g(\mathbf{x}) \rVert}r\right)  $$

where *g*(**x**) represents the gradient and *round* operator rounds each vector element to its nearest integer. On position *P*(*x*), an orientation projection image *O*_*r*_ is increased by 1 and magnitude projection image *M*_*r*_ by ∥*g*(**x**)∥. Transformation is defined as mean over all radii

15 $$ S=\frac{1}{N}\sum_{r\in R} F_{r} \ast G_{r}  $$

where

16 $$ F_{r}(\mathbf{x})=\frac{M_{r}(\mathbf{x})}{k} \left(\frac{|\hat{O}_{r}(\mathbf{x})|}{k} \right)^{\alpha}  $$

17 $$ \hat{O}_{r}(\mathbf{x}) = \left\{ \begin{array}{ll} O_{r}(\mathbf{x}) & \quad \text{if}~ |O_{r}(\mathbf{x})|< k \\ k & \quad otherwise\\ \end{array}\right.  $$

where *G*_*r*_ is a Gaussian kernel, *α* is the radial strictness parameter and *k* is a scaling factor normalizing different radii (where typically *k*≈10). Inspired by Ram et al. [57], we use a gray-scale dilatation to small local maxima suppression in *S*. Local maxima are then considered as cells. As *R* we use all integer values between estimated minimal and maximal cell radius. The parameters for this method include: radial strictness *α*, scaling factor *k*, size of morphology structuring element *δ*, and gradient threshold *β*.

#### Generalized Radial-symmetry transform

The generalized radial-symmetry transform as described by Bahlman et al. [32] (referred as dGRST) is able to deal with elliptical shapes because affine transform is employed.. Similarly to generalized LoG filters, we can compute response for different axis scalings and rotations. The dGRST principle is similar to dFRST method, but the gradient *g*(*x*) is transformed to

18 $$ \hat{g}(\mathbf{x}) = G M G^{-1} M^{-1} {g}(\mathbf{x})  $$

where

19 $$ M= \left[\begin{array}{cc} 0 & 1 \\ -1 & 0 \end{array}\right]  $$

and *G* is affine transformation matrix - for ellipse it is rotation and scaling with parameters *θ*, *a* and *b*. We can set *r*=1 and used *a* and *b* to adjust the size of the desired ellipse axis. All integer values between estimated minimal and maximal cell radius with *a*>*b* and 6 steps for *θ* were used for *a* and *b*. Bahlmann at al. [32] mentioned also a Gaussian kernel specified by affine transformation parameters *θ*, *a* and *b*. For consistency with dFRST, we use Gaussian kernel with *σ*=0.5 distorted with *G* transformation. Remaining parameters are identical to dFRST.

#### Radial voting

Qi et al. [33] presented a modification of radial voting for cells in histopathology specimens (reffered here as dRV-Qi). It is based on an iterative radial voting described previously [58], but works as a single-path voting followed by a mean-shift clustering. Every pixel with position **x**=[*x*,*y*] vote in Gaussian smoothed gradient direction **α**(**x**), with cone shaped-kernel function (voting area).

20 $$ {\begin{aligned} A(x,y,r_{min},r_{max},\Delta) = \left\{x+r cos\phi,y + r sin\phi | r_{min}<\right.\\\quad \left. r< r_{max}, \theta - \Delta<\phi<\theta + \Delta \right\} \end{aligned}}  $$

where *θ* is an angle of vector **α**(**x**), {*r*_*min*_,*r*_*max*_} is kernel radial range and *Δ* is the kernel angular range. In addition, voting sector is weighted by Gaussian function with center located at kernel center. Every pixel (with gradient above certain threshold) update voting image *V* by adding voting pixel gradient magnitude |*g*(**x**)| to all pixels under kernel. Voting image is then thresholded with several thresholds and results are summed and clustered with mean-shift algorithm. For more details see [33]. We used estimated *r*_*min*_ and *r*_*max*_ from the ground truth images, thresholds were set to 0.2, 0.3,...0.9-times the maximum of image, and we optimized sigma of gradient Gaussian smoothing, sigma of Gaussian for kernel and mean shift bandwidth. We also vote with all pixels, not only with pixels with high gradient magnitude, because computational time of our implementation is not dependent on number of voting pixels. Besides [33] we also tested original [58] and newer [59] methods, but both were less suitable for adherent cells.

#### Maximally stable extremal region

Extremal regions of gray-value image are defined as connected components of thresholded image *I*_*t*_=*I*>*t* for some *t* in this method (designated as dMSER). As described in [34], dMSER produces stable extremal regions of image which are stable in sense of area variation w.r.t. changing threshold *t*. Minimal stability of extracted region can be set with two parameters - threshold step *δ* defining the percentage of intensity range and maximal relative area change with this step. This method generates many regions which can overlap. Finally, the smallest regions generated with the highest threshold are picked. This is achieved by finding of the local maxima in the sum of binary images of all regions. Another option is the usage of most stable region from the overlapped ones, but this was shown to be noneffective in our case.

#### CellDetect

Arteta et al. [35] implementation of CellDetect uses MSER to identify the candidate regions, followed by a classification of true regions. Method extracts 92-dimensional feature vector with object histograms and shape descriptors. Training proceeds in two phases. In the first phase, training of binary SVM and its evaluation is done, which produces score for each region. Region with one seed-point and highest score (one for each seed-point) is used as ground truth for the next phase. In the second phase, structured SVM is used for classification of the regions within each tree created from the overlapping regions, but non-overlap constrains are included. For more details see [35]. Method requires few training images with simple dot annotation and proper setting of MSER detector to achieve high recall.

### Single cell (instance) segmentation

After image reconstruction, foreground-background segmentation and seed-point extraction, individual cells were segmented using Marker-controlled (or seeded) watershed [60]. This step showed to be less crucial, because inaccuracy in placing border between cells has a small influence to segmentation efficacy only. Thus, for combining of foreground and seeds into the final segmentation, we test only this simple but very robust technique. Note that more advanced methods exist - e.g. graph-cut [61], or level-set [62] based.

Maker-controlled watershed is similar to classic watershed approach, with restriction of local minima positions into detected seeds location, which can be simply done with mathematical morphology operations. Besides of straightforward application on our images, we proposed a second approach applied on DT image, which does not require an intensity valley between separated cells. For DT image we use geodesic distance transform [63] with distances from seeds (the distance within the foreground pixels only, ignoring the background).

### General parameter optimization strategy

Grid search with 10 steps was used for the optimization of parameters of all methods, where suitable range was selected experimentally by a few manual tests. Parameters with large searched range (relatively large difference between lower and upper bound) were searched with logarithmic scale. The same parameters ranges were used for all modalities. All parameters were properly set for training images and then these values were used for all testing images. For background segmentation and detection methods Dice coefficient was used as an objective function (used e.g. in [18]). For image reconstruction techniques the area under ROC curve (AUC) generated by thresholding was used (as well as in [14] or [12]). Because of large computational difficulty of some methods, we attempted to eliminate such parameters from optimization, which does not influence the objective function. If threshold is optimized parameter, its value was optimized between a minimal and maximal intensity of image pixels, with 100 steps for simplicity. Before application of each method, images were normalized into interval [0,1], where minimal and maximal values of the first image of each sequence were used as a reference for the normalization.

### Evaluation of results

The *F*_1_ score (Dice coefficient) was used as a measure of segmentation accuracy for (1) foreground-background segmentation, (2) seed-point extraction, and (3) single cell segmentation, with following modifications:

#### Foreground-background segmentation evaluation

For the evaluation of cell segmentation, Dice coefficient was used as follows:

21 $$ Dice=\frac{2|X\cap Y|}{|X|+|Y|}  $$

where |·| is number of pixels of region, X and Y are ground truth and result segmentation, respectively. Dice coefficient is equal to *F*_1_-score, but this term is used for pixel-wise evaluation. Another metric used for segmentation evaluation is Jaccard index, which is related to Dice coefficient as:

22 $$ Jaccard=\frac{Dice}{2-Dice}  $$

which is monotonically increasing function on interval <0,1> (the range of Dice values). This means that order of quality of segmentation algorithms w.r.t. Jaccard is same as w.r.t. Dice coefficient and for this reason we evaluated only Dice coefficient.

Dice coefficient was computed for evaluation of the foreground segmentation results using all pixels in the image.

#### Seed-point extraction evaluation

Single dot labels (seeds) are considered as cell detection results. If some method produces pre-segmented regions, then centroids are used as labels. Because our ground truth corresponds to the binary segmented cells, we consider as TP (true positive) such cells having one seed only. As FP (false positive) are considered cells with additional seeds in one cell and with seeds outside cells. FN (false negative) are cells without any seed. To evaluate the performance of the cell detection, Dice coefficient (*F*_1_ score) was used

23 $$ Dice = \frac{2TP}{2TP + FP + FN}.  $$

In some papers the accuracy of the centroid positions is also evaluated. Nevertheless, these positions are not very significant for cell segmentation. Therefore, we didn’t evaluate this accuracy.

#### Single cell segmentation evaluation

For single cell segmentation evaluation the *F*_1_ score (Dice coefficient) is used in a similar manner as for foreground-background segmentation evaluation with following modifications: We dealt with correspondence of objects. We used same evaluation of correspondence as [64] in their SEG evaluation algorithm – cell are considered as matching if:

24 $$ |X\cap Y|>0.5 |X|  $$

which ensures unambiguous pairing. The final measure of Dice was calculated as the mean of the Dice coefficient of all the reference objects. The cells which are on the image boundary were labeled and they are not included in the evaluation.

A computer with following specifications was used to estimate computational times: Intel Core i5-6500 CPU, 8 GB RAM.

## Additional file

Additional file 1 Optimal values for parameters of individual reconstruction methods (xlsx table). * highest value not reducing sensitivity, ** not learned because of identification of small number of regions. nan, not a number. (XLSX 17 kb)

## Abbreviations

AUC: Area under curve
DIC: Differential image contrast
DMGW: Dry mass-guided watershed
DT: Distance transform
EGT: Empirical gradient treshold
FOV: Field of view
FRST: First radial symmetry transform
GRST: Generalized Radial symmetry transform
HMC: Hoffman modulation contrast LoG: Laplacian of Gaussian
MCWS: Marker-controlled watershed
MIP: Maximum intensity projection MSER: maximally stable extremal region
PC: Phase contrast
PD: Poisson distribution
PSF: Point spread function
PT: Poisson treshold
ROC: Receiver-operator curve
RV: Radial voting
ST: Simple treshold
